# Artificial Intelligence in Cardiology: The Current Applications and Future Directions

**DOI:** 10.7759/cureus.99270

**Published:** 2025-12-15

**Authors:** Beka Mikeladze, Giorgi Nikolaishvili, Naira Kobaladze

**Affiliations:** 1 Cardiology, Avicenna-Batumi Medical University, Batumi, GEO; 2 Cardiology, High-Tech Hospital Medcenter, Batumi, GEO; 3 Cardiology, Batumi Shota Rustaveli State University, Batumi, GEO

**Keywords:** ai, cardiology, cvds, machine learning, wearable technology

## Abstract

Artificial intelligence (AI), particularly in its subfields of machine learning and deep learning, is rapidly transforming the landscape of cardiovascular medicine at a very rapid rate. This narrative review provides a concise overview of current applications and prospects of AI in cardiology. The review also examines the central role of AI in predictive analytics and personalized medicine, from forecasting readmission of heart failure to personalizing drug dosing. The review highlights the growing role of wearable devices and telemedicine in enabling remote, near-real-time monitoring of patients. The article concludes by outlining the future of AI in cardiology, noting the need for innovation in explainable AI (XAI), addressing data bias, and establishing robust regulatory frameworks. The integration of AI is not a linear technological progression but a paradigm shift toward precise, efficient, and patient-centric cardiovascular treatment, in which human intelligence and machine intelligence are complementary.

## Introduction and background

Cardiovascular diseases (CVDs) remain the leading causes of global mortality and morbidity [1]. Despite substantial advances in diagnostics and treatments, many clinical workflows remain time-consuming and labor-intensive, with considerable inter-observer variability. The scope and complexity of modern healthcare data, including high-resolution cardiac imaging, comprehensive electronic health records (EHRs), and near-real-time data from wearable devices, now challenge the traditional analytic capacity and consistency of individual clinicians.

This explosion of information has created a rich ground for the application of artificial intelligence (AI). AI encompasses computational methods that learn patterns and generate predictions from complex data, enabling analysis of large, multidimensional datasets and the detection of subtle, clinically relevant signals [2]. In cardiology, that ability is being used to improve every stage of patient management, from initial diagnosis to extended prognosis. Compared with many traditional statistical approaches, modern machine learning methods can capture nonlinear relationships and high-dimensional interactions, revealing patterns that may be missed by conventional analysis. This paradigm shift can potentially transform cardiology from a reactive, population-centered philosophy to an active, highly individualized one, eventually resulting in earlier disease diagnosis, improved treatments, and enhanced patient outcomes [3]. This review summarizes key applications of AI in cardiology, including diagnostic imaging, predictive analytics, and personalized medicine, and outlines the principal challenges and opportunities for clinical integration.

Materials and methods

This review is based on a structured search of PubMed and the Cochrane Library, with supplementary searches in Google Scholar to capture additional relevant literature. The search was conducted using a combination of keywords and Medical Subject Headings (MeSH) to ensure a broad yet focused retrieval of relevant literature. Search terms included "artificial intelligence", "machine learning", "deep learning", "cardiology", "cardiovascular diseases", "ECG", "electrocardiogram", "cardiac imaging", "wearable technology", "telemedicine", and "remote patient monitoring." These terms were used both individually and in various combinations (e.g., "artificial intelligence and cardiology", "deep learning and ECG"). The search covered literature published between 2014 and 2024.

The inclusion criteria for the selected studies were based on their relevance to the application of AI in clinical cardiology, specifically focusing on diagnostic and prognostic models, as well as the use of AI in wearable technology and telemedicine. Only studies published in English were considered. We excluded articles that were not peer-reviewed, were based on purely theoretical models without clinical data, or focused on non-cardiovascular applications. Priority was given to methodologically rigorous studies directly relevant to the review’s themes, with attention to diversity in modality, population, and study design; influential and widely cited works were included to contextualize the field’s evolution. Our initial search yielded a total of 17 articles, from which six were excluded based on the aforementioned criteria, resulting in a final selection of 11 sources for this review.

## Review

Current applications

AI’s impact in cardiology has arguably been most visible in diagnostic data analysis, with notable advances in risk prediction, workflow optimization, and patient monitoring. This section examines representative studies that illustrate AI’s transformative potential.

Enhanced Diagnostics: ECG Analysis and Cardiac Imaging

AI-powered ECG analysis (the atrial fibrillation (AFib) breakthrough): AI has demonstrated an unprecedented ability to recognize subtle patterns in normal 12-lead ECGs that cannot be seen by the naked eye. The most dramatic example of this is the detection of AFib in a normal sinus rhythm. In a landmark study, Attia et al. (2017) trained a deep learning algorithm on over 180,000 ECGs from more than 100,000 patients [4]. The AI algorithm was tasked with finding signs of AFib in ECGs that had already been classified as normal, and predicted AFib in a separate validation set with an area under the curve (AUC) of 0.90.

This degree of accuracy suggested that the AI was detecting a "signature" of AFib when the heart is in normal rhythm, perhaps months or even years before an AFib episode is clinically observed. This finding is in contrast to traditional methods, which are only able to diagnose AFib when AFib is present at diagnosis. The AI algorithm offers the potential for opportunistic screening for AFib, a major cause of stroke, using a simple, widely available, and inexpensive test.

AI in cardiac imaging (improved efficiency and accuracy): Beyond signal analysis, AI is revolutionizing cardiac imaging by automating and enhancing the interpretation of echocardiograms, CT scans, and MRIs. For example, a study from the Mayo Clinic demonstrated a deep learning model capable of quantifying left ventricular ejection fraction (LVEF) from echocardiograms [5]. The model’s LVEF measurements showed a strong correlation with those performed by expert cardiologists, with a high degree of concordance. The significant benefit was not just the accuracy, but the speed; the AI could perform the analysis in seconds, compared to the minutes required for manual measurements by a sonographer. This is particularly valuable in busy clinical settings where time is a critical factor.

Comparative Analysis: AI vs. Traditional Methods

In comparison to conventional approaches, some important benefits of these AI-based diagnostic tools are:

(i) Speed and efficiency: AI algorithms can process thousands of images or ECGs while an individual expert reads one, bringing an enormous reduction in workflow bottlenecks.

(ii) Reduced variability: Unlike human interpreters, whose interpretations can vary considerably, AI models provide objective and reproducible measurements with a reduction in inter-observer variability.

(iii) New discoveries: As the study by Attia et al. [4] discloses, AI can recognize hidden patterns and anticipate states that are beyond the reach of traditional diagnostic methods, thereby offering new avenues for early intervention and prevention.

Predictive Analytics and Precision Medicine

AI’s role extends beyond diagnostics into predicting disease trajectories and personalizing treatment.

Predicting heart failure readmission: Heart failure is one of the leading causes of hospital readmission, which places a great burden on patients and healthcare systems. A study by Golas et al. used an AI model to predict 30-day readmission risk for patients with heart failure [6]. By analyzing a complex mix of structured data from EHRs (e.g., lab results, medications, comorbidities) and unstructured data (e.g., clinician notes), the model achieved a predictive accuracy that outperformed conventional, simpler risk scores like the LACE (Length of stay, Acuity of admission, Comorbidity, and Emergency department (ED) use) index. AI’s ability to process and find correlations within diverse data sources allowed it to identify at-risk patients with greater precision, enabling targeted interventions and reducing preventable readmissions.

Personalized warfarin dosing: The anticoagulant warfarin has a slender therapeutic index, and finding the appropriate dose in a patient can be difficult due to genetic and lifestyle variation. It is often required to employ a trial-and-error strategy with consecutive blood analyses. For the improvement of this process, Choi et al. developed a machine learning model to predict the optimal initial dose of warfarin [7]. This model integrated clinical variables (age, weight, height) and genetic data (CYP2C9 and VKORC1 genotypes) to provide a more precise dosage recommendation. Predictions of the model led to better outcomes and reduced adverse events compared to conventional dosing regimens.

These examples highlight AI’s shift from a simple diagnostic tool to a sophisticated partner in clinical decision-making, offering insights that are vital for both improving patient care and optimizing healthcare resource utilization.

The Rise of Wearable Technology in Cardiovascular Monitoring

The popular adoption of wearable devices, particularly smartwatches, has transformed the paradigm of cardiovascular monitoring from intermittent to real-time. They possess enhanced sensors such as photoplethysmography (PPG) for rhythm and rate measurement and built-in ECG capabilities. This near-real-time monitoring generates an unprecedented volume of longitudinal data previously feasible only in clinical settings.

One of the clearest examples is AFib detection using smartwatches. In the Apple Heart Study, a prospective trial with 419,297 participants, a smartwatch-based algorithm detected irregular pulse patterns suggestive of AFib [8]. Participants who received notifications were sent a wearable ECG patch for validation. Among analyzable ECGs, 34% confirmed AFib, with a positive predictive value of 0.84. This study demonstrates the potential of wearables as a mass-screening tool for asymptomatic AFib, a silent but significant predictor of stroke.

Moreover, wearable devices are increasingly evolving beyond simple rhythm detection. Recent research and reviews highlight how data from these devices can be integrated with AI algorithms to predict individual cardiovascular risk, including the development of hypertension. For example, an American College of Cardiology publication emphasizes that combining wearable technology with AI enables real-time monitoring and early intervention, allowing both clinicians and patients to act proactively rather than relying solely on episodic clinic visits [9]. This shift facilitates continuous, individualized surveillance, ultimately improving patient outcomes and supporting preventive cardiovascular care.

Telemedicine and Remote Patient Monitoring: Expanding the Reach of AI

In addition to the clinic and wearables, AI plays a central role in the rapidly expanding field of telemedicine and remote patient monitoring (RPM). RPM is the application of digital technology to collect health data from patients in their homes and electronically send that information to healthcare providers. AI algorithms are required to sort through this constant stream of data, highlighting the subtle trends that could signal an emerging problem. 

A seminal study by Stevenson et al. explored the use of remote monitoring to track heart failure patients' weight, symptoms, and activity levels [10]. The system aimed to predict impending decompensation and potential hospitalization. Although the study did not show a reduction in hospitalization rates, it highlighted the potential of continuous, AI-assisted monitoring to identify early signs of deterioration, enabling clinicians to intervene preemptively through medication adjustments or lifestyle guidance, ultimately supporting more proactive heart failure management.

A strong example of AI-driven remote monitoring in hypertension management comes from recent studies. Acharya et al. demonstrated that remote patient monitoring, including daily blood pressure tracking, was associated with reduced mortality and hospitalization rates among hypertensive patients [11]. Complementing this, Lee et al. reviewed innovative AI-based platforms for remote hypertension management, highlighting their potential to identify patients with consistently elevated readings or poor medication adherence and to streamline clinical workflows [12]. Together, these findings underscore the ability of AI and wearable technologies to enhance blood pressure control, support proactive interventions, and improve patient outcomes compared with standard care.

The integration of AI with telemedicine marks a shift from reactive “sick care” to proactive “well care.” Instead of responding only to acute conditions, this approach focuses on the continuous management of individual health. AI’s capacity to analyze vast datasets from thousands of patients, identify those at highest risk, and alert clinicians in real-time is transformative. This enables not only personalized and proactive care but also scalable solutions, particularly benefiting rural or underserved populations with limited access to advanced healthcare services.

Future directions and challenges

The future of AI in cardiology holds immense promise, yet several significant challenges must be addressed for its full potential to be realized. The integration of AI into clinical practice presents technological, ethical, regulatory, reimbursement, and logistical challenges.

Advancements in Personalized and Predictive Medicine

The next major frontier for AI lies in predictive and highly personalized medicine. Future algorithms will move beyond merely estimating risk to creating individualized therapeutic maps. Imagine a scenario in which AI integrates a patient’s full genetic profile, real-time data from sensors, and lifestyle information from wearables to predict not only the likelihood of a cardiovascular event, such as a heart attack, but also its timing and an optimized treatment plan to prevent it. Researchers are already developing AI-based platforms capable of predicting a patient’s response to specific medications, assisting physicians in selecting the most effective drug with minimal side effects. Such approaches have the potential to substantially reduce the reliance on trial-and-error methods that remain prevalent in pharmacology [13].

The "Black Box" Problem and XAI

One of the most pressing challenges in AI integration into healthcare is the "black box" problem, where complex deep learning algorithms provide outputs without transparent reasoning. This opacity can erode clinician trust, hinder accountability, and impede informed decision-making. To address these issues, the field of XAI has emerged, aiming to develop models that are both accurate and interpretable. Recent studies highlight that enhancing transparency in AI systems is crucial for fostering trust among healthcare professionals and ensuring ethical decision-making in patient care [14].

Ethical Considerations and Data Integrity

The broad application of AI in cardiology introduces significant ethical considerations. The effectiveness of an AI model is heavily influenced by the diversity and quality of its training data. If a dataset is unbalanced, such as being predominantly from a specific demographic, the resulting model may not only perform suboptimally but also perpetuate and exacerbate existing health disparities. Ensuring equitable representation in datasets and conducting rigorous external validation across diverse patient populations are essential steps to mitigate algorithmic bias. Furthermore, safeguarding patient data security and privacy is paramount. Robust regulatory frameworks and clear ethical guidelines are necessary to develop and deploy AI technologies responsibly in healthcare [15,16].

Clinical Integration and Physician-AI Collaboration

One of the main future directions for AI in cardiology is in workflow integration. Despite its excellent performance in data processing, human oversight remains irreplaceable. Ultimately, the best model for future cardiovascular care might be one in which AI systems support clinicians but do not substitute for them [17]. Such integration can reduce cognitive load, minimize diagnostic variability, and enhance efficiency [18]. However, this transformation requires structured physician training, continual algorithm auditing, and the establishment of adaptive clinical protocols that ensure AI recommendations are interpreted in the appropriate clinical context.

Data Infrastructure, Interoperability, and Global Equity

In equal measure, the development of robust data infrastructure and interoperability between health systems is important. Access to standardized, high-quality datasets reflecting a diverse population and healthcare environment is crucial for the functionality of AI tools [19]. Collaboration in international data sharing may reduce biases and accelerate innovation [20]. Further, broadening the adoption of AI in low- and middle-income settings will prevent further exacerbation of the global health equity gap. Sustainable, ethics-guided implementation will help distribute the benefits from AI-driven cardiology equitably and globally.

## Conclusions

The use of AI in cardiology is no longer a theoretical concept but a reality that is beginning to transform clinical practice at its foundation. From automating the nuanced interpretation of ECGs and cardiac imaging to enabling ongoing remote monitoring via smartwatches and telemedicine, AI has demonstrated its potential in transitioning cardiovascular care from a reactive to an active, preventive model. The predictive prowess of AI, illustrated here by its capacity to help forecast heart failure readmissions and support individualized drug dosing with accuracy, augurs the arrival of truly personalized medicine.

Even though significant challenges remain, not least the need for integrative algorithmic transparency, de-biasing of data, and the creation of stringent ethical and regulatory frameworks, but the way forward is clear. AI does not signify a replacement for the human cardiologist but a powerful adjunct that will improve the standard of care. The future of cardiology will be marked by the cooperation of human intelligence and machine intelligence, working with one another to diagnose sooner, treat more precisely, and ultimately, improve the lives of patients worldwide.

## References

[1] World Health Organization. (2025, July 31). Cardiovascular diseases (2025). World Health Organization: Cardiovascular diseases (CVDs). https://www.who.int/news-room/fact-sheets/detail/cardiovascular-diseases-%28cvds%29.

[2] Liu J, Cen X, Yi C (2025). Challenges in AI-driven biomedical multimodal data fusion and analysis. Genomics Proteomics Bioinformatics.

[3] Shu S, Ren J, Song J (2021). Clinical application of machine learning-based artificial intelligence in the diagnosis, prediction, and classification of cardiovascular diseases. Circ J.

[4] Attia ZI, Noseworthy PA, Lopez-Jimenez F (2019). An artificial intelligence-enabled ECG algorithm for the identification of patients with atrial fibrillation during sinus rhythm: a retrospective analysis of outcome prediction. Lancet.

[5] Attia IZ, Tseng AS, Benavente ED (2021). External validation of a deep learning electrocardiogram algorithm to detect ventricular dysfunction. Int J Cardiol.

[6] Golas SB, Shibahara T, Agboola S (2018). A machine learning model to predict the risk of 30-day readmissions in patients with heart failure: a retrospective analysis of electronic medical records data. BMC Med Inform Decis Mak.

[7] Choi H, Kang HJ, Ahn I (2023). Machine learning models to predict the warfarin discharge dosage using clinical information of inpatients from South Korea. Sci Rep.

[8] Perez MV, Mahaffey KW, Hedlin H (2019). Large-scale assessment of a smartwatch to identify atrial fibrillation. N Engl J Med.

[9] American College of Cardiology. (2025, July 1 (2025). American College of Cardiology: The future of digital health in cardiovascular disease: bridging innovation and clinical practice. https://www.acc.org/Latest-in-Cardiology/Articles/2025/07/01/01/Cover-Story-The-Future-of-Digital-Health-in-CVD#resources-for-article.

[10] Stevenson LW, Ross HJ, Rathman LD, Boehmer JP (2023). Remote monitoring for heart failure management at home. J Am Coll Cardiol.

[11] Acharya M, Ali MM, Bogulski CA, Pandit AA, Mahashabde RV, Eswaran H, Hayes CJ (2024). Association of remote patient monitoring with mortality and healthcare utilization in hypertensive patients: a Medicare claims-based study. J Gen Intern Med.

[12] Lee SG, Fisher ND (2023). Innovative remote management solutions for the control of hypertension. Hypertension.

[13] Zack M, Stupichev DN, Moore AJ, Slobodchikov ID, Sokolov DG, Trifonov IF, Gobbs A (2025). Artificial intelligence and multi-omics in pharmacogenomics: a new era of precision medicine. Mayo Clin Proc Digit Health.

[14] Marey A, Arjmand P, Alerab AD, Eslami MJ, Saad AM, Sanchez N, Umair M (2024). Explainability, transparency and black box challenges of AI in radiology: impact on patient care in cardiovascular radiology. Egypt J Radiol Nucl Med.

[15] Chin MH, Afsar-Manesh N, Bierman AS (2023). Guiding principles to address the impact of algorithm bias on racial and ethnic disparities in health and health care. JAMA Netw Open.

[16] Hasanzadeh F, Josephson CB, Waters G, Adedinsewo D, Azizi Z, White JA (2025). Bias recognition and mitigation strategies in artificial intelligence healthcare applications. NPJ Digit Med.

[17] Topol EJ (2019). High-performance medicine: the convergence of human and artificial intelligence. Nat Med.

[18] Davenport T, Kalakota R (2019). The potential for artificial intelligence in healthcare. Future Healthc J.

[19] Benjamens S, Dhunnoo P, Meskó B (2020). The state of artificial intelligence-based FDA-approved medical devices and algorithms: an online database. NPJ Digit Med.

[20] Panch T, Mattie H, Atun R (2019). Artificial intelligence and algorithmic bias: implications for health systems. J Glob Health.

